# Supplementary material for: Degradation of Benzotriazole UV Stabilizers in PAA/d-Electron Metal Ions Systems—Removal Kinetics, Products and Mechanism Evaluation
Source: Molecules. 2022 May 23;27(10):3349. doi: 10.3390/molecules27103349 (PMC9145517; doi:10.3390/molecules27103349)
Supplement: Supplementary file 1 [file molecules-27-03349-s001.zip › molecules-1730610-supplementary.pdf]

molecules

Supplementary Information

Degradation of benzotriazole UV stabilizers in PAA/d-electron metal ions systems–removal kinetics, products and mechanism evaluation

Dariusz Kiejza [1], Joanna Karpińska [2] and Urszula Kotowska [2,*]

[1] Doctoral School of Exact and Natural Sciences, University of Bialystok, Ciołkowskiego 1K St., 15-245 Białystok, Poland; d.kiejza@uwb.edu.pl
[2] Department of Analytical and Inorganic Chemistry, Faculty of Chemistry, University of Bialystok, Ciołkowskiego 1K St., 15-245 Białystok, Poland; joasia@uwb.edu.pl (JK), ukrajew@uwb.edu.pl (UK)
* Correspondence: ukrajew@uwb.edu.pl; Tel.: +48 85 738 8111

Table S1. The chemical structure, chemical abstract service (CAS) registry number, molecular weights (MW), octanol-water partition coefficients (log Kow), acid dissociation constants (pKa), quantification and identification ions (m/z) of target benzotriazole UV stabilizers.

Systematic name, other names	Structure	CAS number	MW (g/mol)	log K_{ow}	pKa	Quantification and identification ions (m/z)[a]
**UV-P**<br>drometrizole, Tinuvin P	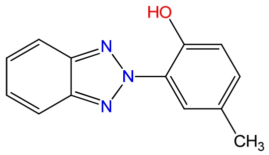	2440-22-4	225.25	4.2	8.15	168, 196, **225**
**UV-326**<br>bumetrizole, Tinuvin 326	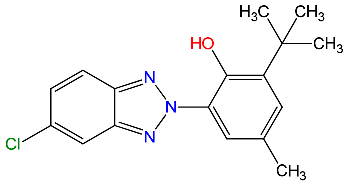	3896-11-5	315.8	5.52	9.49	272, **300**, 315
**UV-327**<br>Tinuvin 327	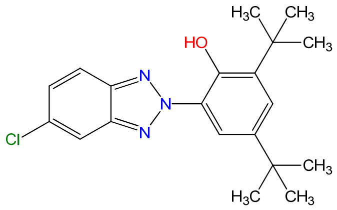	3864-99-1	357.88	6.75	9.41	314, **342**, 357

**UV-328**<br>Tinuvin 328	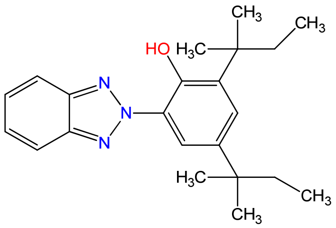	25973-55-1	351.49	7.22	8.73	**322**, 336, 351
**UV-329**<br>octrizole, Tinuvin 329	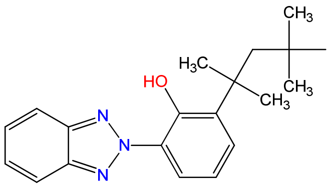	3147-75-9	323.44	6.2	–	**252**, 224, 323

[a] bolded ions selected for quantification.

Synthesis of peracetic acid

Peracetic acid (PAA) was synthesized according to procedure described by [1]. 10 mL of glacial acetic acid was placed in a ground glass bottle. Then 0.94 mL of 95 % sulfuric acid was added. In the next step, 10 ml of 30 % hydrogen peroxide was carefully added dropwise. The bottle was placed in an ice bath and continuously mixed with a magnetic stirrer for 90 minutes. After this time the PAA concentration is about 6 %. After 48 hours, when an equilibrium between the substrates and products is established, 16 % peracetic acid is reached. PAA was stored at 4 °C for no more than a month. PAA working solutions were prepared by dilution in deionized water and stored at 4 °C for no longer than 2 days.

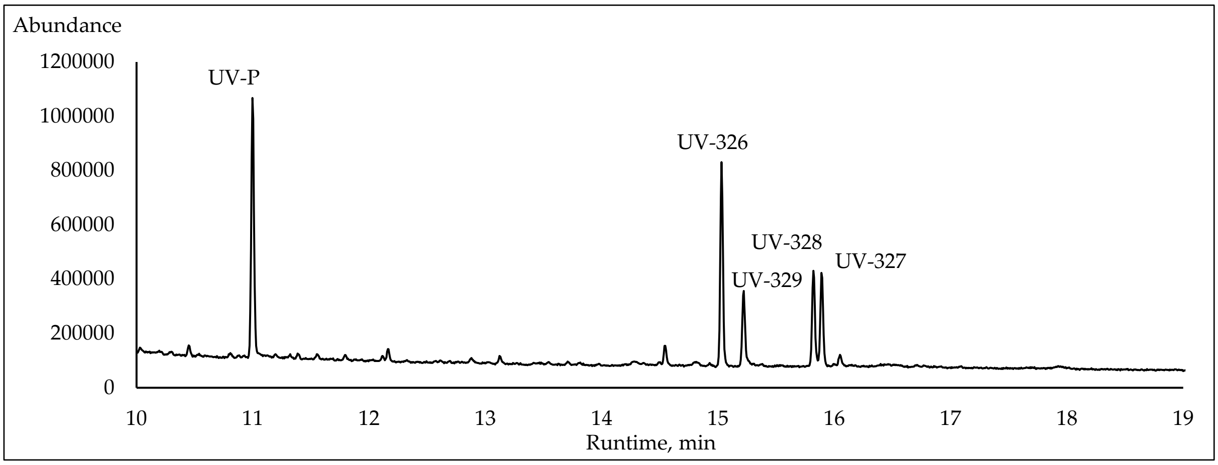

Figure S1. Chromatogram of the studied benzotriazole UV stabilizers.

UV-P

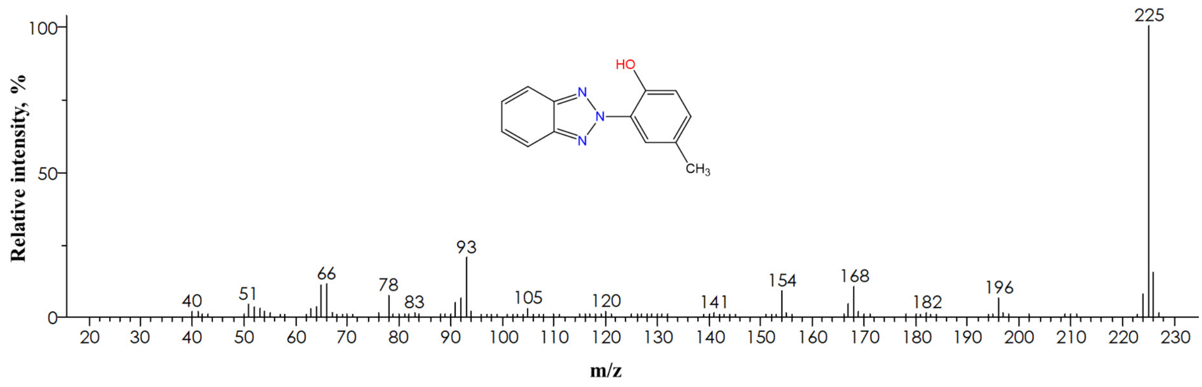

UV-326

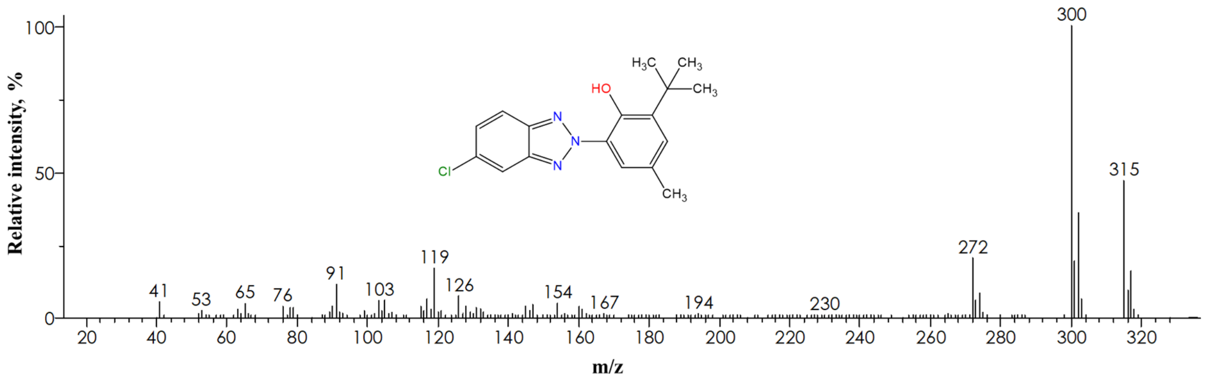

UV-327

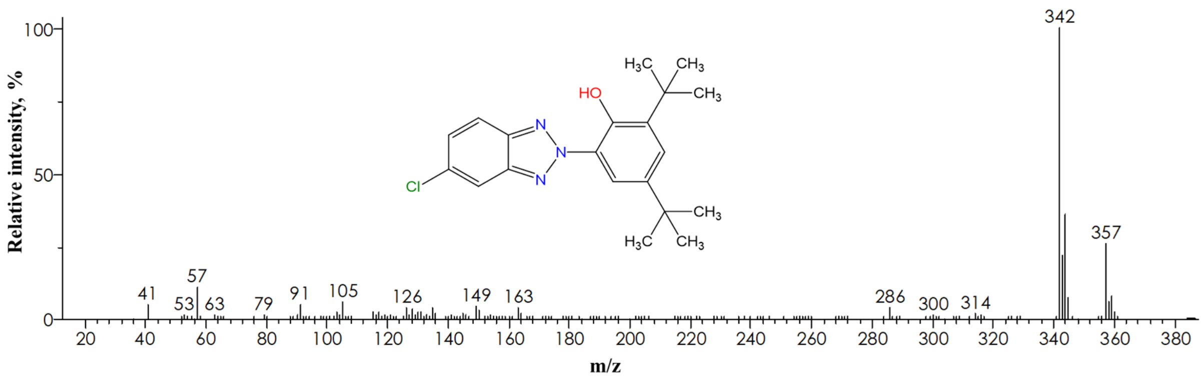

UV-328

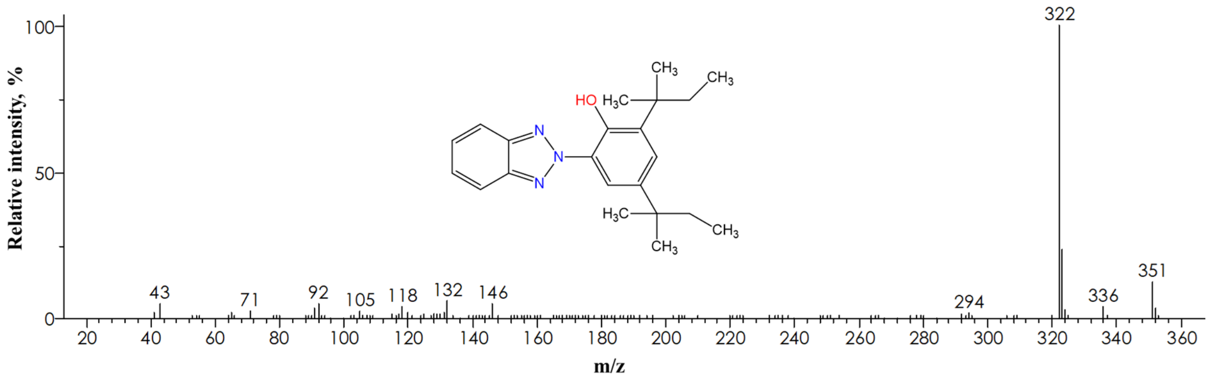

UV-329

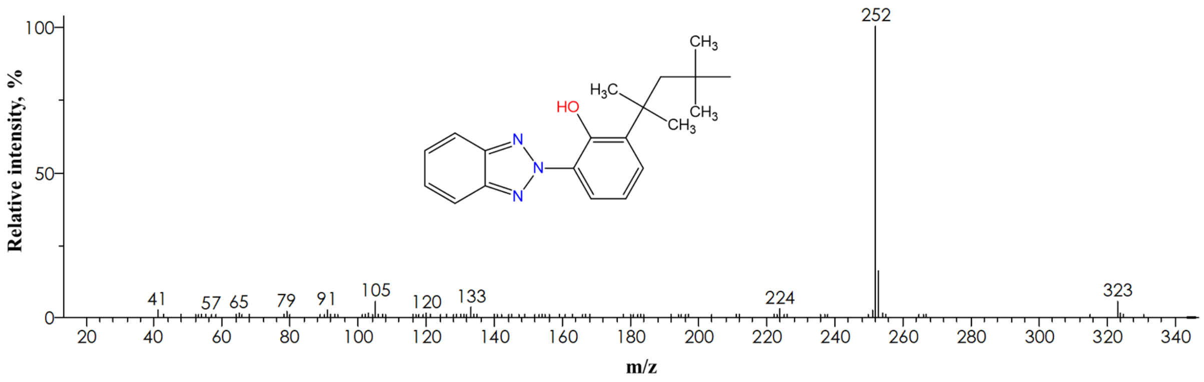

Figure S2. Mass spectra recorded during GC-MS analysis of studied compounds.

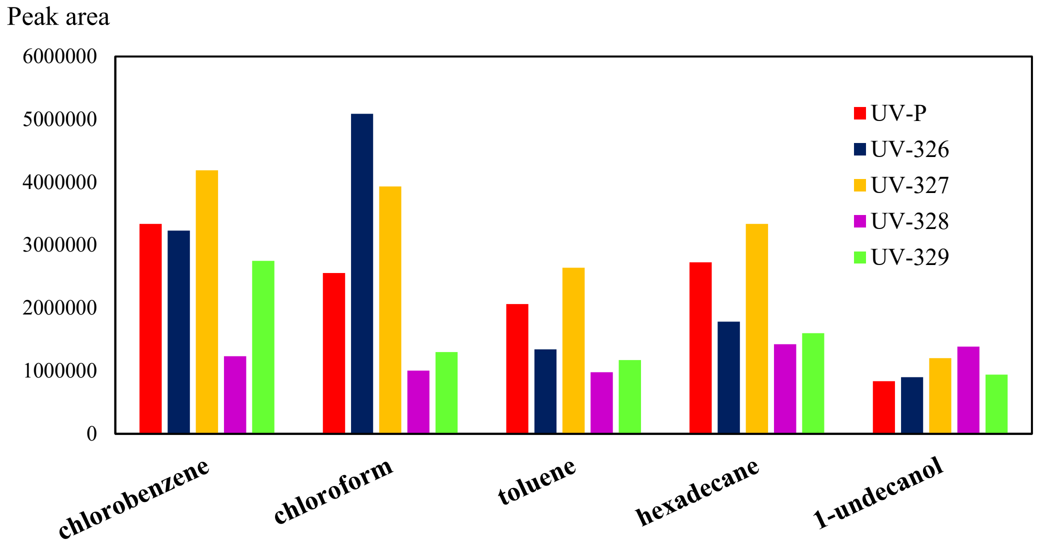

Figure S3. Comparison of the UV stabilizers peak areas depending on the different extractants.

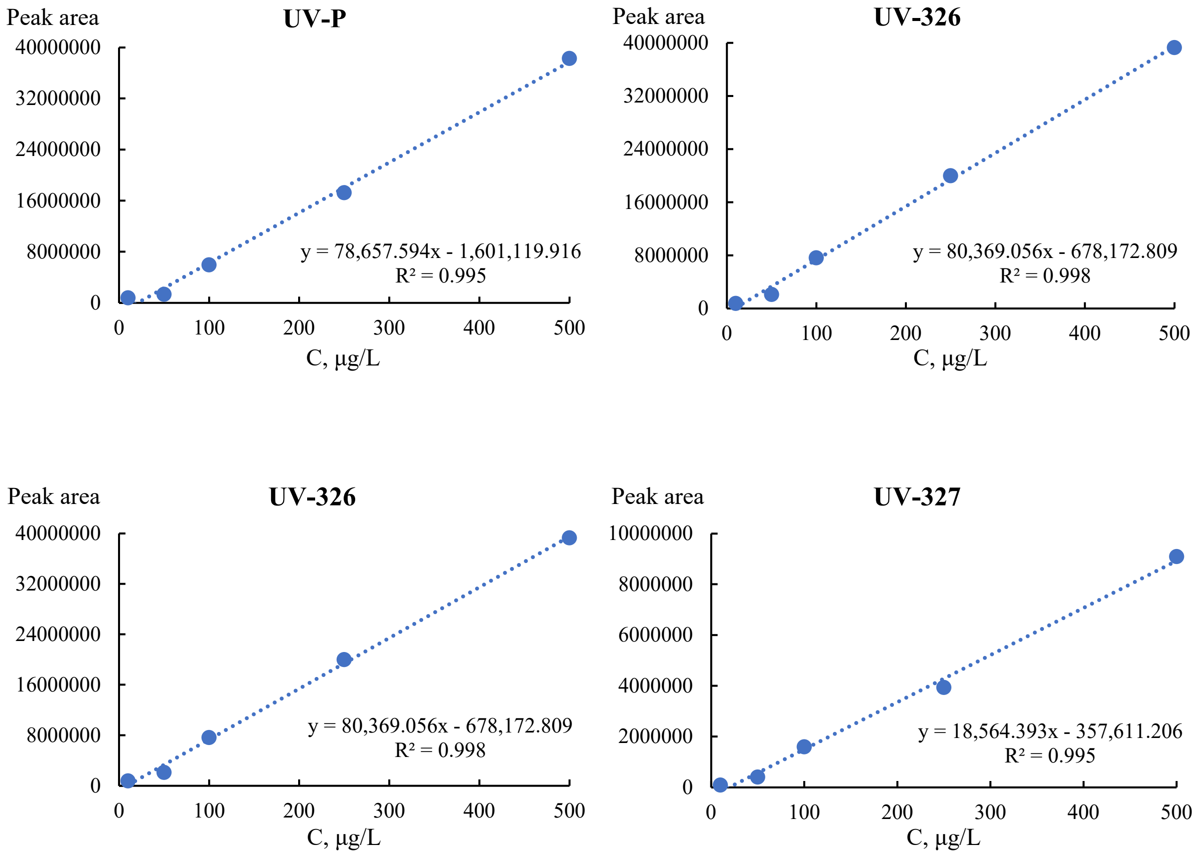

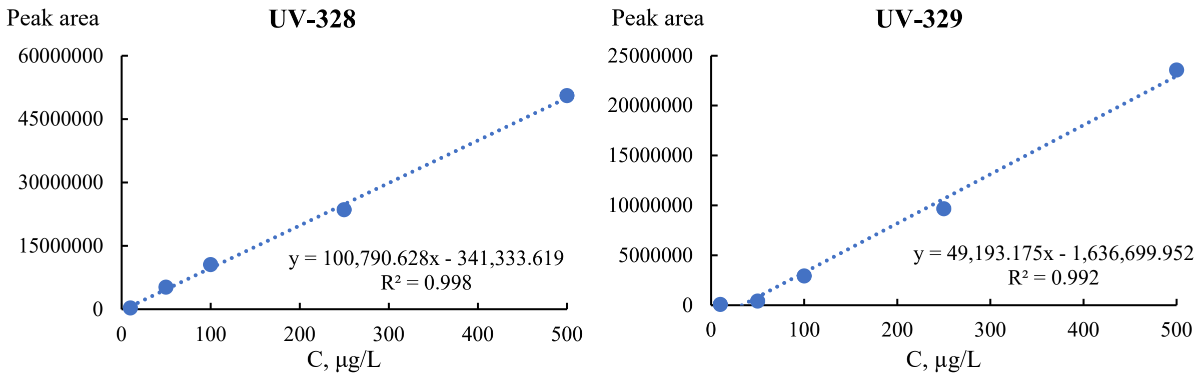

Figure S4. Calibration curves used to calculate temporary benzotriazole concentrations.

Table S2. Experimental and predicted removal efficiency values for benzotriazole UV stabilizers degradation in Fe^{2+}/PAA process.

Run	$[PAA]_0$ (mg/L)	$[Me^{2+}]_0$ (mol/L)	pH	UV-P		UV-326		UV-327		UV-328		UV-329	
				Exp.	Pred.	Exp.	Pred.	Exp.	Pred.	Exp.	Pred.	Exp.	Pred.
1	45	$3.45 \cdot 10^{-4}$	4.6	82.86	78.75	57.35	63.37	28.61	29.45	28.61	29.45	70.44	76.42
2	45	$3.45 \cdot 10^{-4}$	3.4	98.01	100.00	76.25	77.84	37.41	37.92	37.41	37.92	80.50	73.06
3	45	$1.45 \cdot 10^{-5}$	4.6	98.76	100.00	73.69	72.96	53.90	44.36	53.90	44.36	80.80	84.41
4	45	$1.45 \cdot 10^{-5}$	3.4	92.80	83.59	55.32	60.78	33.39	36.43	33.39	36.43	58.32	76.78
5	15	$3.45 \cdot 10^{-4}$	4.6	45.62	51.20	66.91	64.61	44.08	44.05	44.08	44.05	70.02	77.33
6	15	$3.45 \cdot 10^{-4}$	3.4	95.29	90.72	75.48	79.08	31.60	36.01	31.60	36.01	72.29	73.97
7	15	$1.45 \cdot 10^{-5}$	4.6	97.29	96.09	75.22	74.20	50.80	51.11	50.80	51.11	80.80	85.31
8	15	$1.45 \cdot 10^{-5}$	3.4	89.39	92.16	49.54	62.02	18.54	26.66	18.54	26.66	50.11	77.69
9	55	$7 \cdot 10^{-5}$	4	95.52	97.90	67.55	66.21	35.68	39.68	35.68	39.68	74.00	78.33
10	5	$7 \cdot 10^{-5}$	4	94.80	93.05	65.49	68.28	46.12	39.36	46.12	39.36	74.52	79.84
11	30	$1 \cdot 10^{-3}$	4	88.42	88.34	65.51	64.61	43.00	48.98	24.81	24.23	74.41	73.83

12	30	$5 \cdot 10^{-6}$	4	96.80	100.00	55.44	66.56	35.31	26.58	27.25	41.57	75.54	80.85
13	30	$7 \cdot 10^{-5}$	5	88.59	85.19	64.78	76.62	25.38	41.29	43.00	48.98	82.29	86.63
14	30	$7 \cdot 10^{-5}$	3	73.20	77.22	58.76	63.78	47.31	41.29	35.31	26.58	76.34	75.12
15	30	$7 \cdot 10^{-5}$	4	98.13	98.01	69.46	67.68	45.78	41.29	25.38	41.29	85.20	79.40
16	30	$7 \cdot 10^{-5}$	4	98.64	98.01	77.01	67.68	47.83	41.29	47.31	41.29	92.07	79.40
17	30	$7 \cdot 10^{-5}$	4	98.70	98.01	77.37	67.68	52.05	41.29	45.78	41.29	93.66	79.40
18	30	$7 \cdot 10^{-5}$	4	99.02	98.01	77.74	67.68	45.25	41.29	47.83	41.29	94.78	79.40
19	30	$7 \cdot 10^{-5}$	4	98.96	98.01	81.38	67.68	43.00	48.98	52.05	41.29	95.96	79.40
20	30	$7 \cdot 10^{-5}$	4	98.62	98.01	76.76	67.68	35.31	26.58	45.25	41.29	93.89	79.40

Table S3. Experimental and predicted removal efficiency for benzotriazole UV stabilizers degradation in Co^{2+}/PAA process.

Run	$[PAA]_0$ (mg/L)	$[Me^{2+}]_0$ (mol/L)	pH	UV-P		UV-326		UV-327		UV-328		UV-329	
				Exp.	Pred.	Exp.	Pred.	Exp.	Pred.	Exp.	Pred.	Exp.	Pred.
1	45	$3.45 \cdot 10^{-4}$	7	70.20	79.37	91.01	100.00	87.55	94.98	83.48	99.61	94.67	100.00
2	45	$3.45 \cdot 10^{-4}$	4	89.77	85.73	94.13	97.24	93.56	100.00	91.72	96.20	96.62	100.00
3	45	$1.45 \cdot 10^{-5}$	7	54.13	58.22	36.99	55.25	15.66	45.78	6.57	39.82	33.31	64.98
4	45	$1.45 \cdot 10^{-5}$	4	59.23	59.14	33.35	46.27	16.56	32.86	1.68	15.87	29.48	42.76
5	15	$3.45 \cdot 10^{-4}$	7	85.54	81.17	97.44	100.00	96.76	100.00	95.63	100.00	99.02	100.00
6	15	$3.45 \cdot 10^{-4}$	4	84.20	93.85	92.99	97.24	91.62	99.49	88.71	100.00	96.44	100.00
7	15	$1.45 \cdot 10^{-5}$	7	51.35	53.38	20.10	55.25	29.03	60.73	3.93	46.71	18.51	53.44
8	15	$1.45 \cdot 10^{-5}$	4	50.43	60.63	23.84	46.27	6.21	30.37	1.27	22.76	7.93	46.03
9	55	$7 \cdot 10^{-5}$	5.5	62.92	63.13	78.68	66.76	72.79	55.22	65.47	43.55	84.80	67.85
10	5	$7 \cdot 10^{-5}$	5.5	63.46	62.20	93.51	66.76	91.97	65.61	89.83	55.03	95.84	63.27
11	30	$1 \cdot 10^{-3}$	5.5	91.24	90.20	90.15	87.04	89.17	85.50	85.99	81.42	95.27	90.88

12	30	$5 \cdot 10^{-6}$	5.5	47.16	62.40	41.04	53.29	28.61	44.82	6.82	40.05	42.89	66.02
13	30	$7 \cdot 10^{-5}$	8	59.55	56.19	93.50	61.48	95.14	62.39	95.84	52.93	95.97	61.30
14	30	$7 \cdot 10^{-5}$	3	70.75	64.52	57.71	46.51	49.23	31.89	32.57	18.76	63.93	40.24
15	30	$7 \cdot 10^{-5}$	5.5	74.57	69.30	75.96	66.76	70.50	60.42	73.76	58.71	93.41	81.15
16	30	$7 \cdot 10^{-5}$	5.5	74.34	69.30	76.79	66.76	71.85	60.42	73.56	58.71	93.03	81.15
17	30	$7 \cdot 10^{-5}$	5.5	75.35	69.30	71.59	66.76	67.83	60.42	76.02	58.71	94.01	81.15
18	30	$7 \cdot 10^{-5}$	5.5	76.23	69.30	72.02	66.76	66.99	60.42	66.46	58.71	92.13	81.15
19	30	$7 \cdot 10^{-5}$	5.5	73.35	69.30	72.57	66.76	74.71	60.42	67.58	58.71	93.33	81.15
20	30	$7 \cdot 10^{-5}$	5.5	72.16	69.30	78.93	66.76	68.29	60.42	67.68	58.71	93.98	81.15

Table S4. ANOVA results for UV-P removal process in Fe^{2+}/PAA system.

**Source of variation**	**Sum of squares**	**DF**	**Mean square**	***F*-value**	***p*-value**
PAA (square)	231.39	1	231.39	111.35	0.815095
Fe^{2+} (linear)	1829.66	1	1829.66	880.51	0.668419
Fe^{2+} (square)	761.42	1	761.42	366.42	0.664894
pH (linear)	141.74	1	141.74	68.21	0.197092
pH (square)	159.86	1	159.86	76.93	0.645401
PAA-Fe^{2+} interactions	41.21	1	41.21	19.83	0.549233
PAA-pH interactions	90.68	1	90.68	43.64	0.269201
Fe^{2+}-pH interactions	17.44	1	17.44	8.39	0.237537
Lack of fit	534.35	6	89.06		
Pure error	10.39	5	2.08		
Total	3221.50	19			
R^2 =0.831	R^2 (adjusted) = 0.708				

Table S5. ANOVA results for UV-326 removal process in Fe^{2+}/PAA system.

**Source of variation**	**Sum of squares**	**DF**	**Mean square**	***F*-value**	***p*-value**
Fe^{2+} (linear)	6283.22	1	6283.22	694.32	0.668419
Fe^{2+} (square)	3527.49	1	3527.49	389.80	0.664894
pH (linear)	273.44	1	273.44	30.22	0.197092
pH (square)	285.90	1	285.90	31.59	0.645401
Lack of fit	5116.54	10	511.65		
Pure error	45.25	5	9.05		
Total	12106.62	19			
R^2 =0.574	R^2 (adjusted) = 0.460				

Table S6. ANOVA results for UV-327 removal process in Fe^{2+}/PAA system.

Source of variation	Sum of squares	DF	Mean square	*F*-value	*p*-value
PAA (linear)	131.71	1	131.71	15.46	0.011055
Fe^{2+} (linear)	8463.52	1	8463.52	993.16	0.000001
Fe^{2+} (square)	4649.28	1	4649.28	545.56	0.000003
pH (linear)	418.03	1	418.03	49.05	0.000914
pH (square)	309.06	1	309.06	36.27	0.001816
PAA-pH interactions	152.00	1	152.00	17.84	0.008301
Fe^{2+}-pH interactions	234.34	1	234.34	27.50	0.003343
Lack of fit	6330.90	7	904.41		
Pure error	42.61	5	8.52		
Total	16607.25	19			
R^2 =0.616	R^2 (adjusted) = 0.392				

Table S7. ANOVA results for UV-328 removal process in Fe^{2+}/PAA system.

**Source of variation**	**Sum of squares**	**DF**	**Mean square**	***F*-value**	***p*-value**
PAA (linear)	160.77	1	160.77	9.74	0.026201
PAA (square)	152.78	1	152.78	9.26	0.028646
Fe^{2+} (linear)	11562.53	1	11562.53	700.86	0.000001
Fe^{2+} (square)	6686.23	1	6686.23	405.29	0.000006
pH (linear)	573.54	1	573.54	34.76	0.001996
pH (square)	899.23	1	899.23	54.51	0.000717
Fe^{2+}-pH interactions	248.96	1	248.96	15.09	0.011587
Lack of fit	9937.66	7	1419.67		
Pure error	82.49	5	16.50		
Total	23717.25	19			
R^2 =0.577	R^2 (adjusted) = 0.492				

Table S8. ANOVA results for UV-329 removal process in Fe^{2+}/PAA system.

Source of variation	Sum of squares	DF	Mean square	*F*-value	*p*-value
PAA (square)	738.90	1	738.90	1530.51	0.000000
Fe^{2+} (linear)	7553.60	1	7553.60	15646.09	0.000000
Fe^{2+} (square)	5010.49	1	5010.49	10378.43	0.000000
pH (linear)	212.72	1	212.72	440.61	0.000005
pH (square)	1663.65	1	1663.65	3445.99	0.000000
PAA-Fe^{2+} interactions	76.59	1	76.59	158.64	0.000056
PAA-pH interactions	15.93	1	15.93	32.99	0.002243
Fe^{2+}-pH interactions	99.22	1	99.22	205.53	0.000030
Lack of fit	8731.99	6	1455.33		
Pure error	2.41	5	0.483		
Total	17929.83	19			
R^2 =0.513	R^2 (adjusted) = 0.492				

Table S9. ANOVA results for UV-P removal process in Co^{2+}/PAA system.

Source of variation	Sum of squares	DF	Mean square	*F*-value	*p*-value
PAA (linear)	275.92		275.92	2786.71	0.000000
PAA (square)	11.06	1	11.06	111.71	0.000131
Co^{2+} (linear)	413.76	1	413.76	4178.89	0.000000
Co^{2+} (square)	167.60	1	167.60	1692.75	0.000000
pH (linear)	284.97	1	284.97	2878.08	0.000000
pH (square)	485.35	1	485.35	4901.92	0.000000
PAA-Co^{2+} interactions	231.92	1	231.92	2342.32	0.000000
PAA-pH interactions	132.66	1	132.66	1339.87	0.000000
Co^{2+}-pH interactions	1114.41	1	1114.41	11255.24	0.000000
Lack of fit	265.26	5	52.85		
Pure error	0.49	5	0.10		
Total	3046.31	19			
R^2=0.913	R^2 (adjusted) = 0.835				

Table S10. ANOVA results for UV-329 removal process in Co^{2+}/PAA system.

Source of variation	Sum of squares	DF	Mean square	*F*-value	*p*-value
PAA (square)	801.37	1	801.37	54.21	0.000726
Co^{2+} (square)	214.78	1	214.78	14.53	0.012478
pH (square)	466.27	1	466.27	31.54	0.002476
Co^{2+}-pH interactions	497.30	1	497.30	33.64	0.002147
Lack of fit	997.67	10	99.77		
Pure error	73.91	5	14.78		
Total	2747.51	19			
R^2 =0.610	R^2 (adjusted) = 0.506				

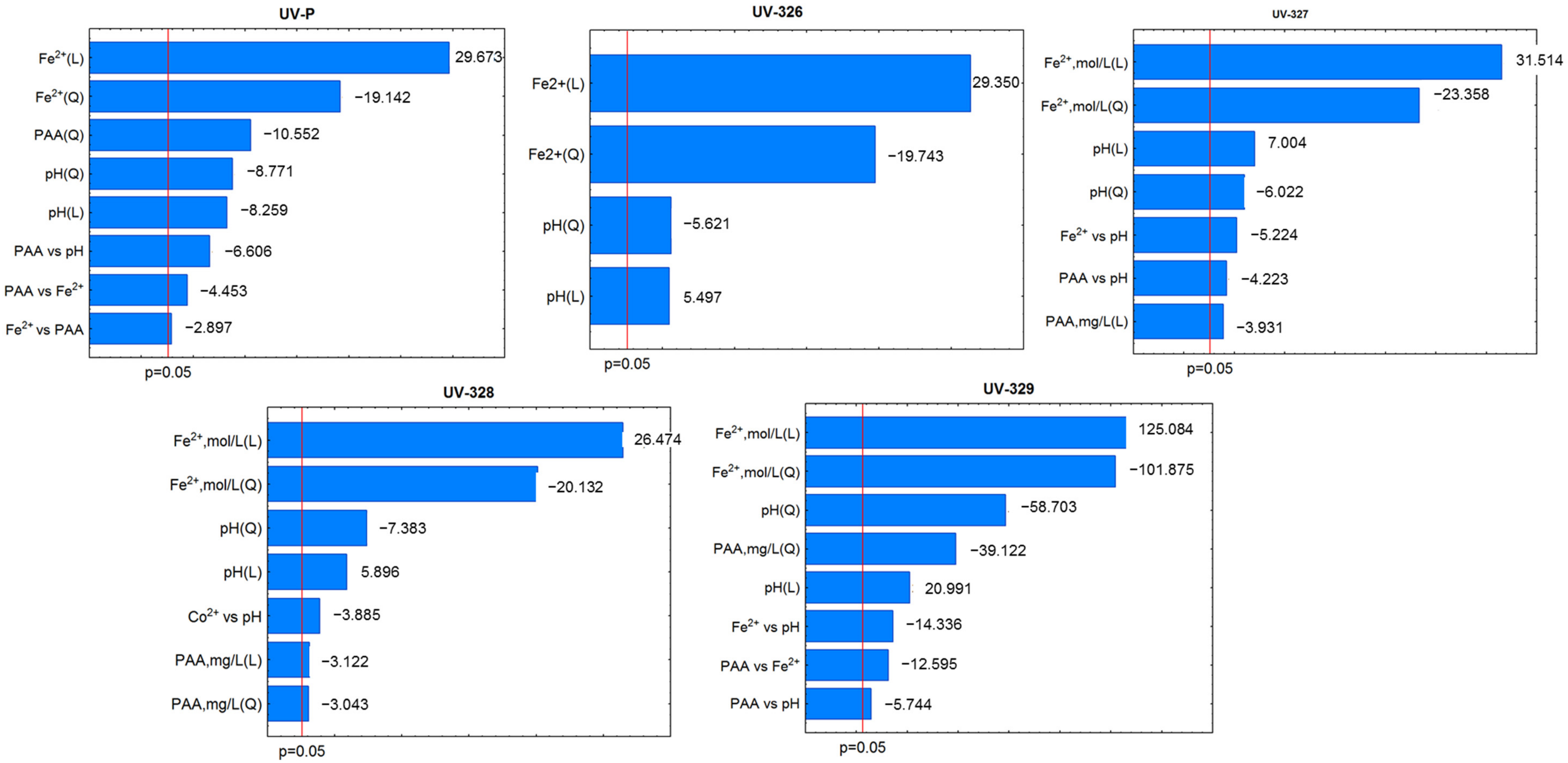

Figure S5. Pareto charts showing the influence of factors and their interactions on the individual BUVs removal efficiency in the Fe^{2+}/PAA process.

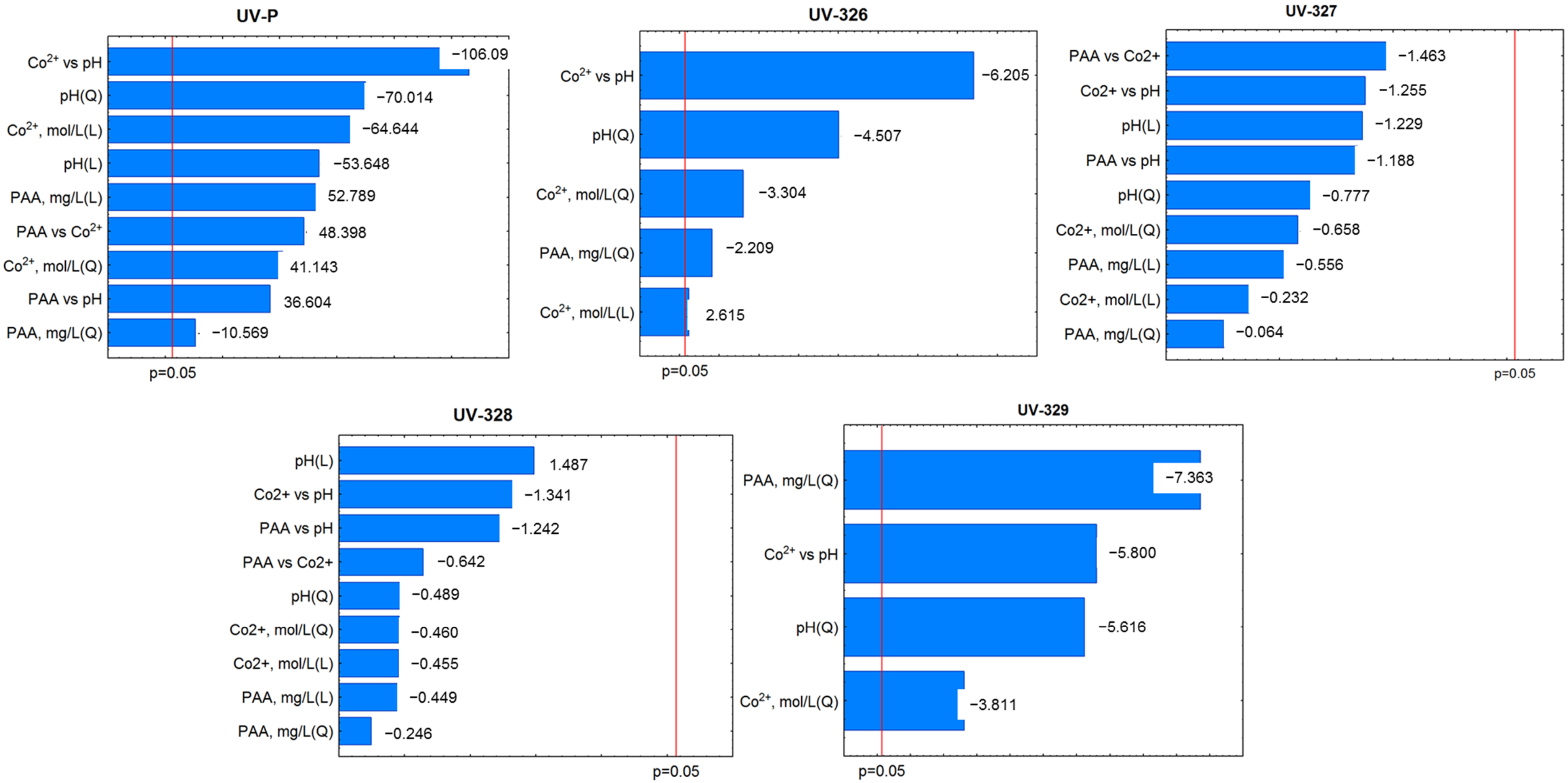

Figure S6. Pareto charts showing the influence of factors and their interactions on the individual BUVs removal efficiency in the Co2+/PAA process.

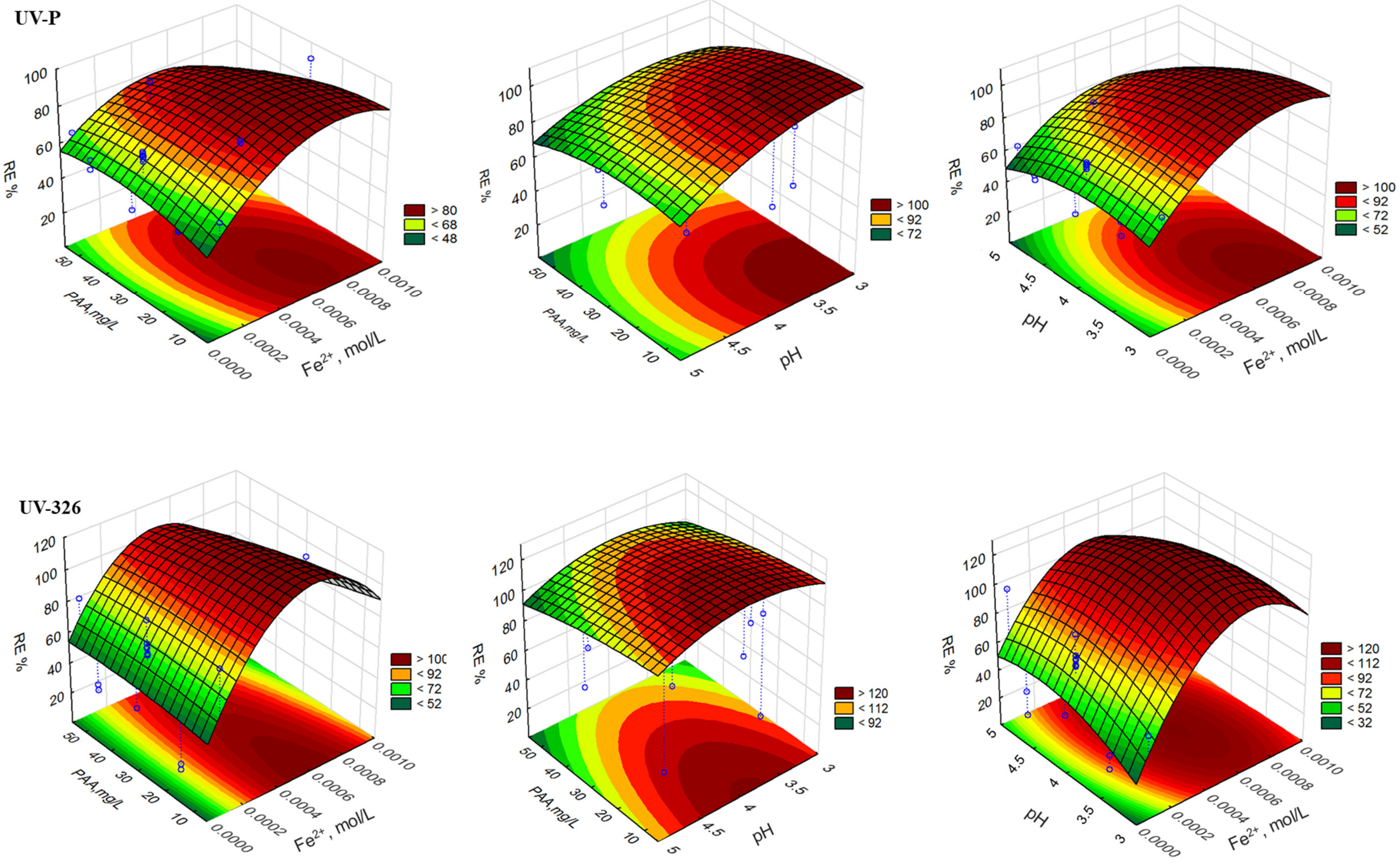

UV-P
RE %
PAA,mg/L
Fe2+, mol/L
pH
> 80
< 68
< 48
> 100
< 92
< 72
> 100
< 92
< 72
< 52
UV-326
> 100
< 92
< 72
< 52
> 120
< 112
< 92
> 120
< 112
< 92
< 72
< 52
< 32

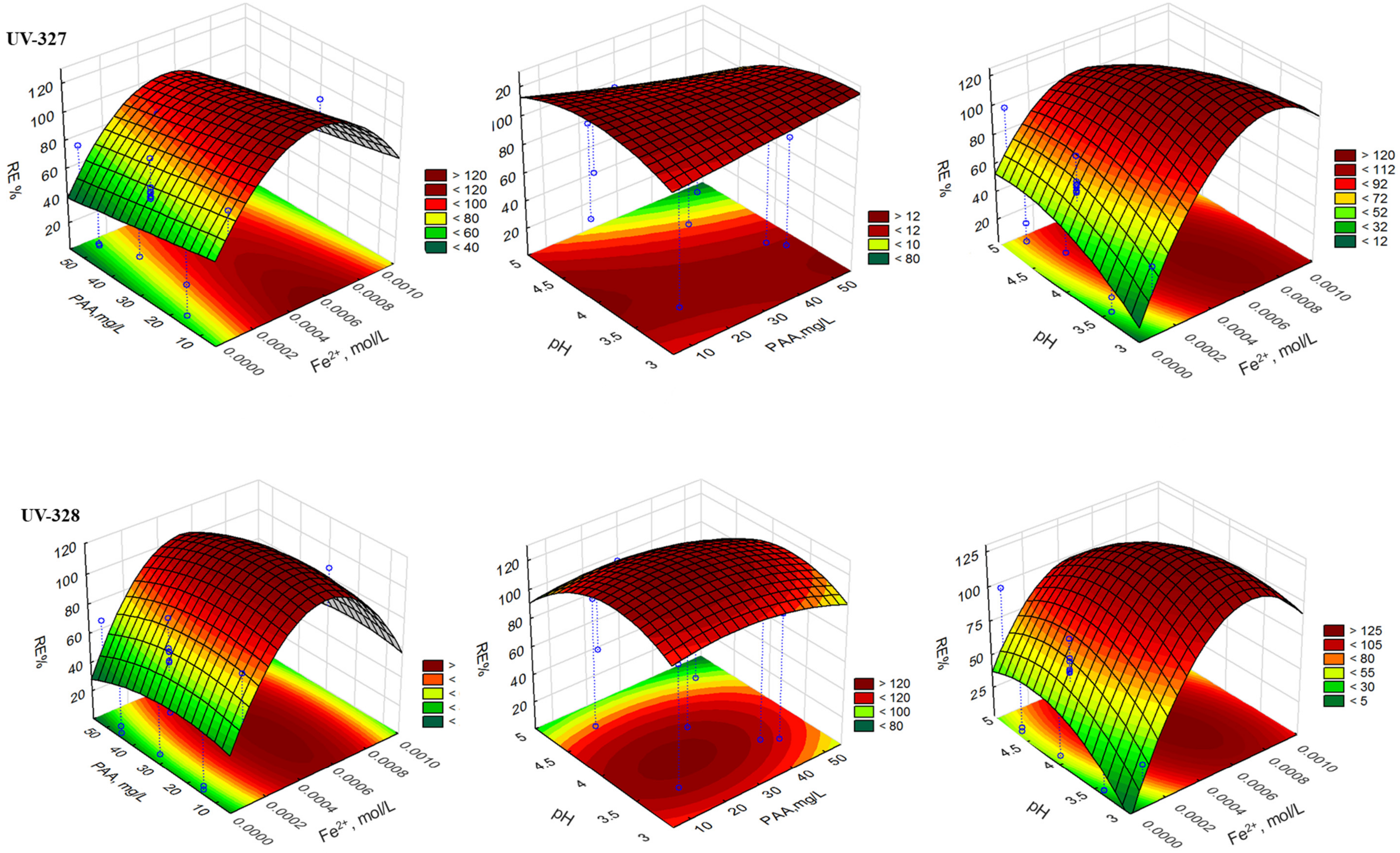
UV-327
RE %
PAA,mg/L
Fe2+, mol/L
> 120
< 120
< 100
< 80
< 60
< 40
pH
PAA,mg/L
> 12
< 12
< 10
< 80
RE %
pH
Fe2+, mol/L
> 120
< 112
< 92
< 72
< 52
< 32
< 12
UV-328
RE%
PAA, mg/L
Fe2+, mol/L
RE%
pH
PAA,mg/L
> 120
< 120
< 100
< 80
RE%
pH
Fe2+, mol/L
> 125
< 105
< 80
< 55
< 30
< 5

UV-329

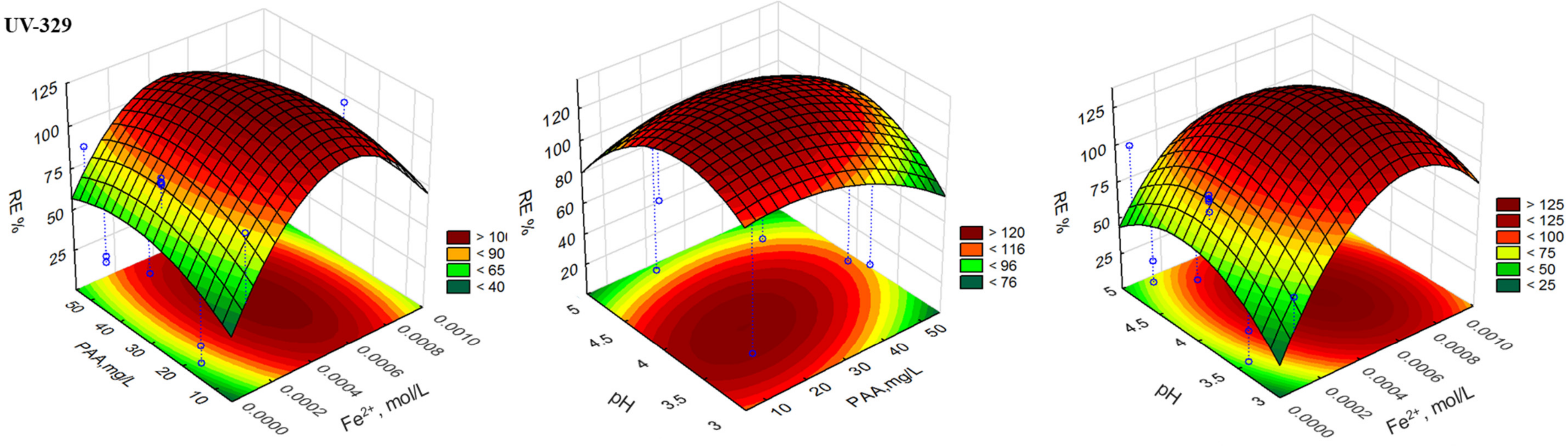

Figure S7. Response surface plots of the removal efficiency of studied BUVs in the Fe^{2+}/PAA process. Conditions: $[PAA]_0$= 25 mg/L, $[Fe^{2+}]_0$= $6 \cdot 10^{-4}$ mol/L, pH=4.5.

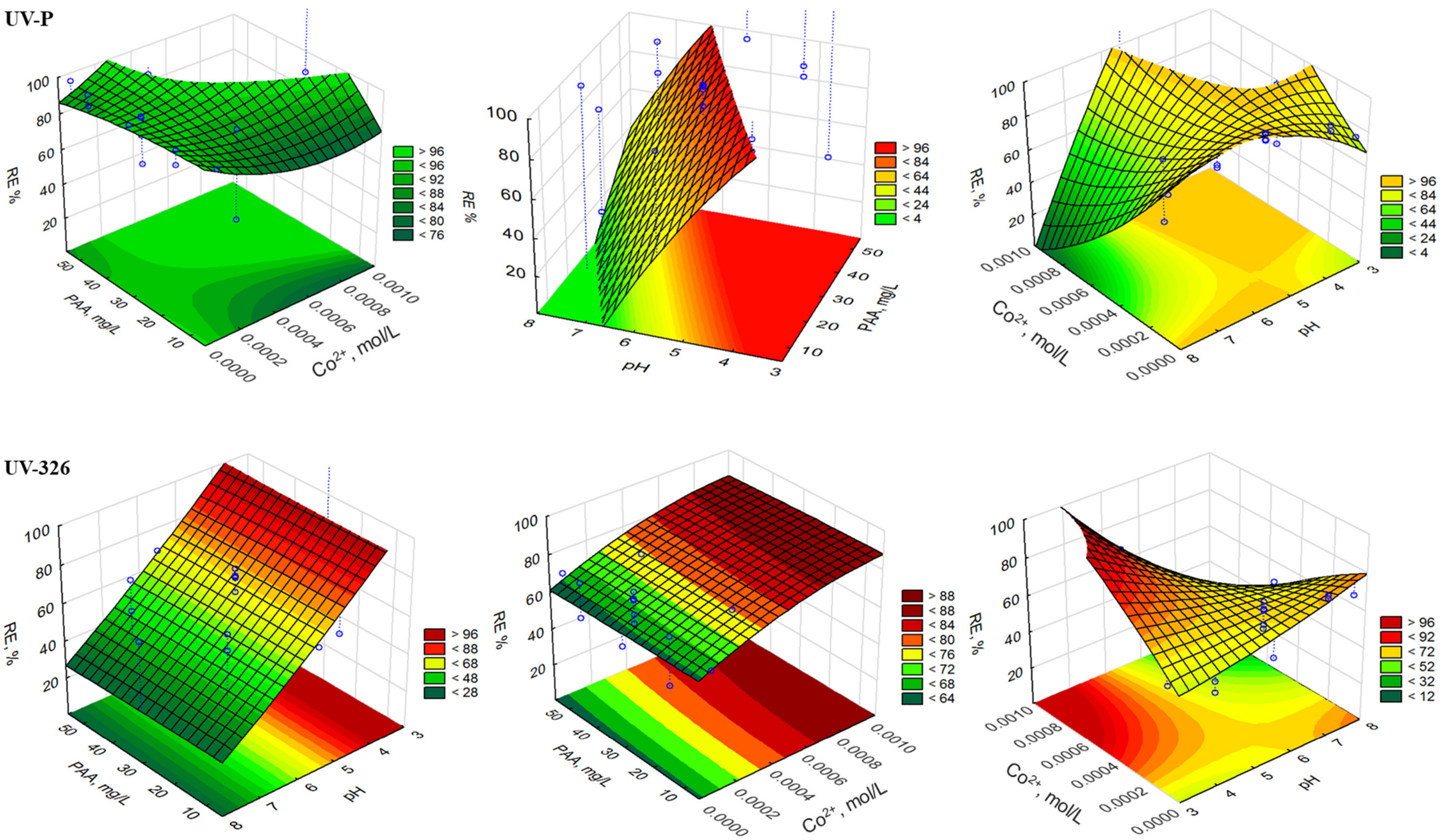
UV-P
RE %
PAA, mg/L
Co2+, mol/L
> 96
< 96
< 92
< 88
< 84
< 80
< 76
pH
> 96
< 84
< 64
< 44
< 24
< 4
UV-326
RE, %
> 96
< 88
< 68
< 48
< 28
> 88
< 84
< 80
< 76
< 72
< 68
< 64
< 92
< 72
< 52
< 32
< 12

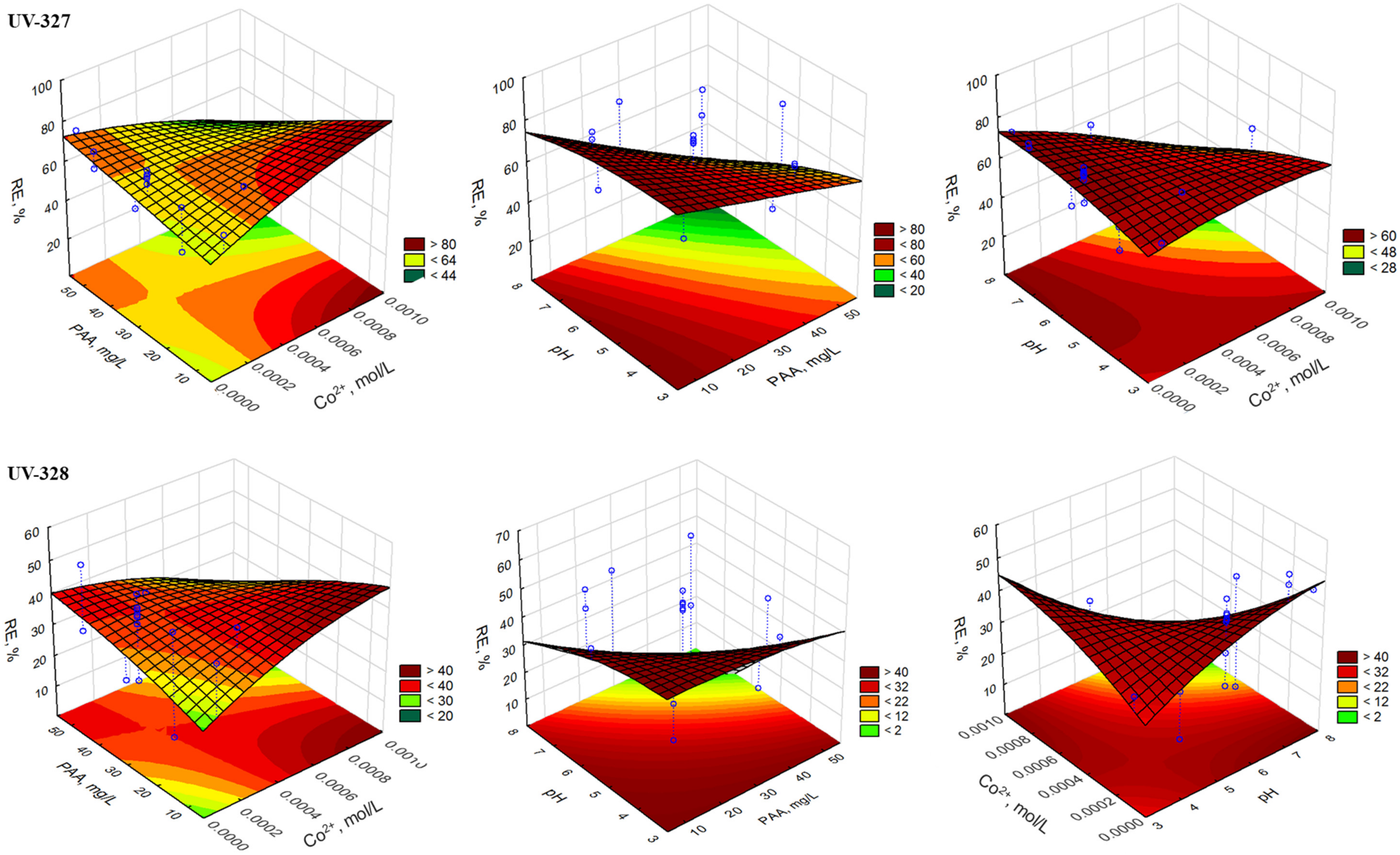

UV-327
UV-328
RE, %
PAA, mg/L
Co2+, mol/L
pH
> 80
< 64
< 44
> 80
< 80
< 60
< 40
< 20
> 60
< 48
< 28
> 40
< 40
< 30
< 20
> 40
< 32
< 22
< 12
< 2

UV-329

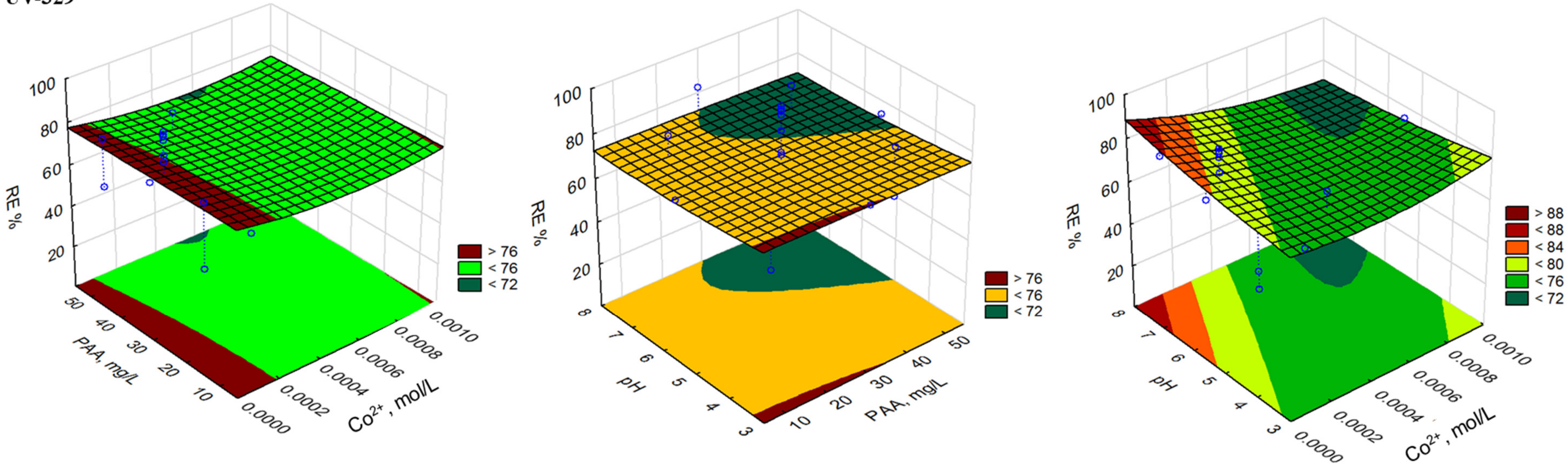

Figure S8. Response surface plots of the removal efficiency of studied BUVs in the Co^{2+}/PAA process. Conditions: $[PAA]_0$= 40 mg/L, $[Co^{2+}]_0$= $8{\cdot}10^{-4}$ mol/L, pH=4.5.

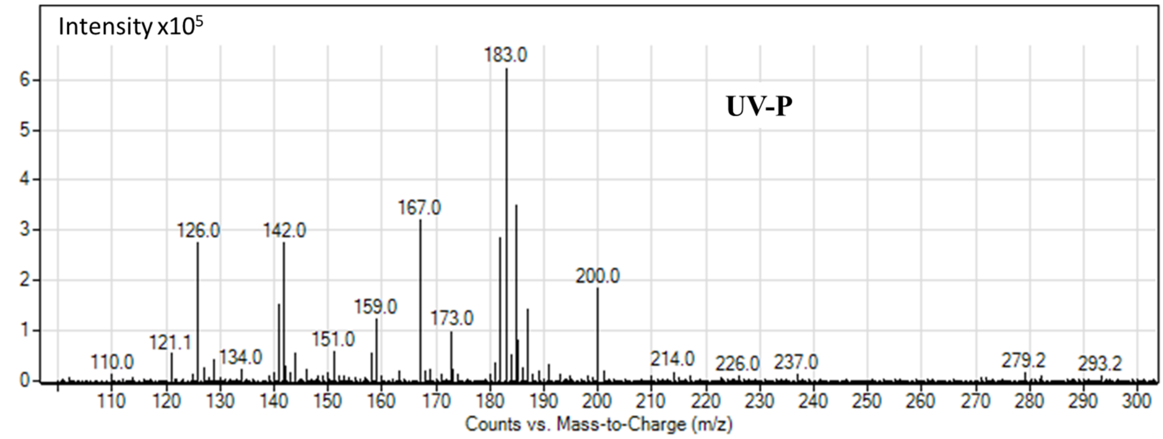
Intensity x10^5
UV-P
183.0
167.0
126.0
142.0
200.0
159.0
173.0
151.0
121.1
134.0
110.0
214.0
226.0
237.0
279.2
293.2
Counts vs. Mass-to-Charge (m/z)

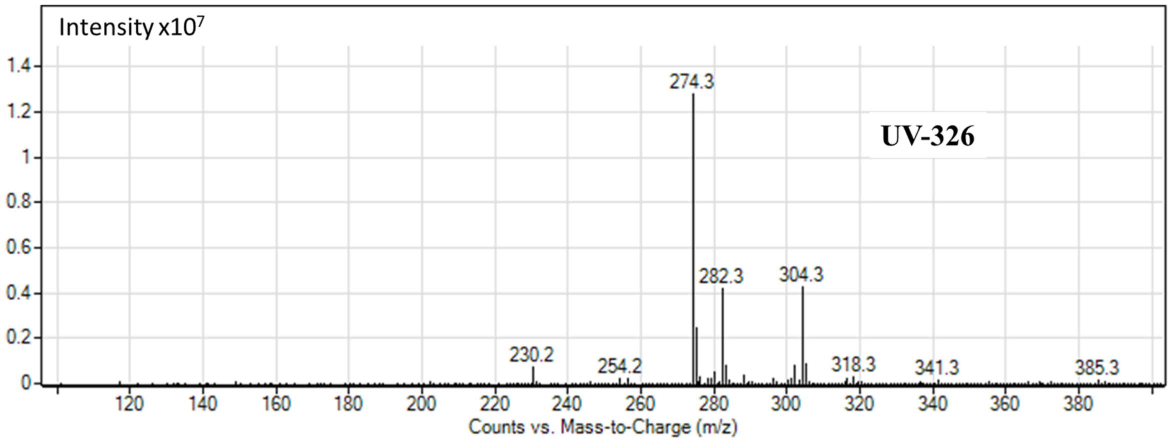
Intensity x10^7
UV-326
274.3
282.3
304.3
230.2
254.2
318.3
341.3
385.3
Counts vs. Mass-to-Charge (m/z)

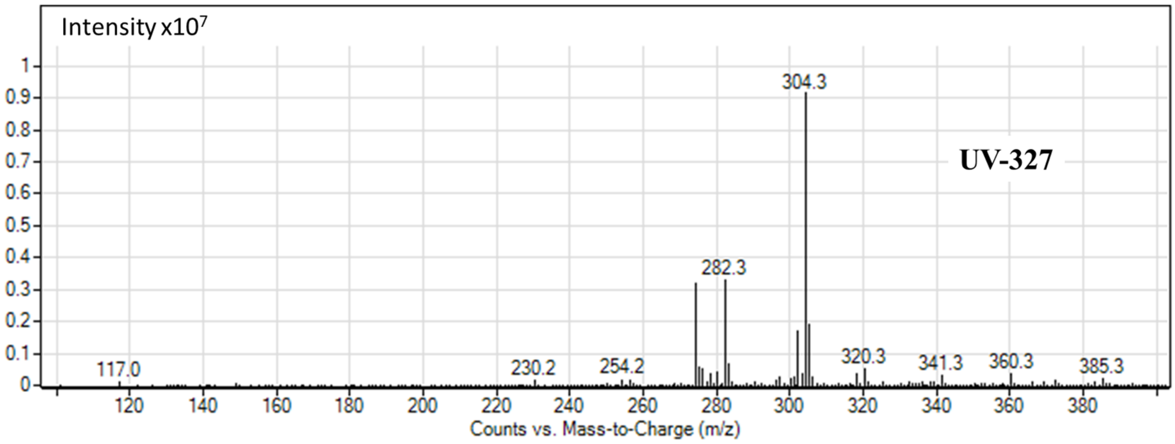
Intensity x10^7
UV-327
304.3
282.3
117.0
230.2
254.2
320.3
341.3
360.3
385.3
Counts vs. Mass-to-Charge (m/z)

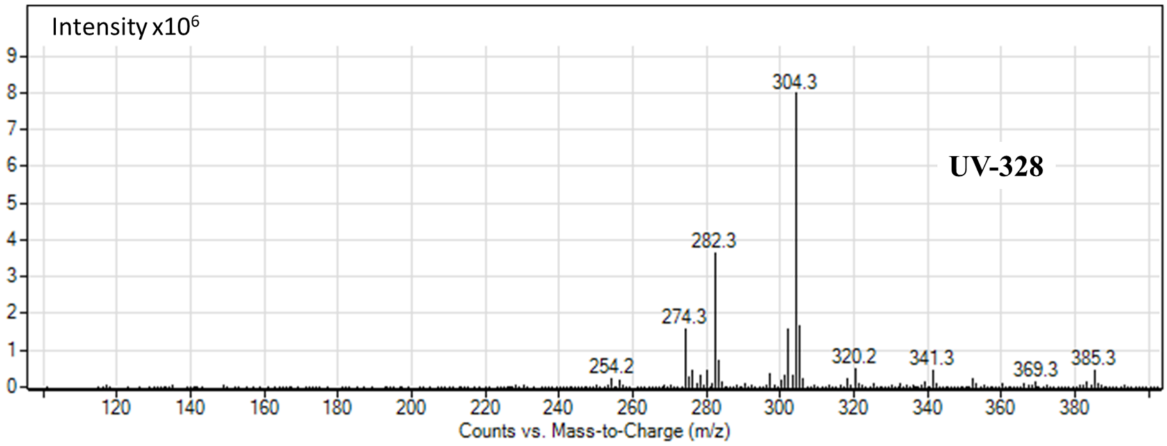
Intensity x10^6
UV-328
304.3
282.3
274.3
254.2
320.2
341.3
369.3
385.3
Counts vs. Mass-to-Charge (m/z)

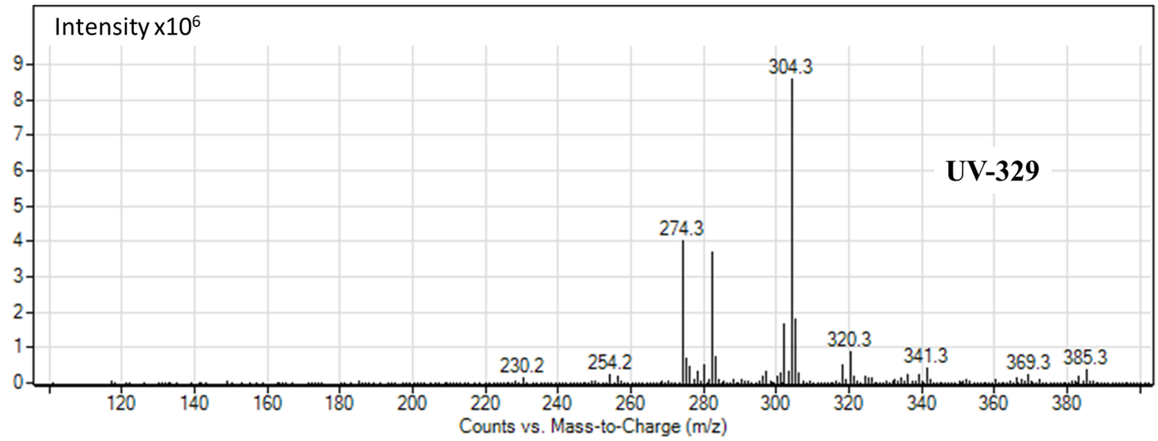

Figure S9. Mass spectra of post-reaction mixture.

Table S11. Proposed structures of benzotriazole UV stabilizers oxidation products.

m/z	Proposed structure	m/z	Proposed structure	m/z	Proposed structure
119	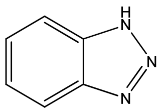<br>Found in: UV-P, UV-328	153	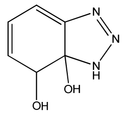<br>Found in: UV-327, UV-328	171	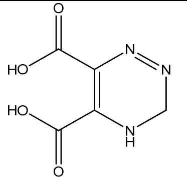 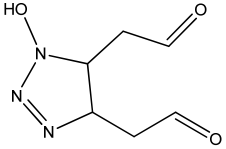<br>Found in: UV-P
125	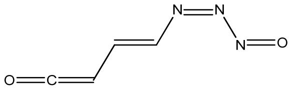<br>Found in: UV-P, UV-328	157	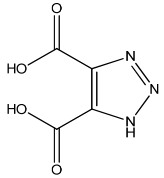<br>Found in: UV-328	175	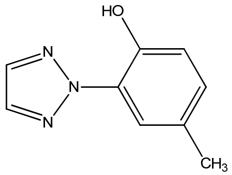<br>Found in: UV-P, UV-326
131	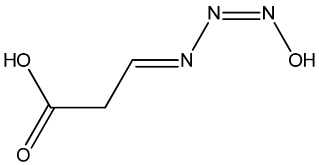<br>Found in: UV-P	158	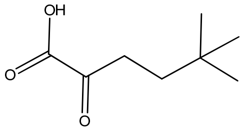<br>Found in: UV-327	181	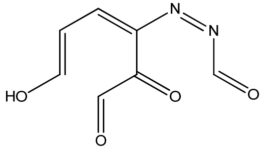<br>Found in: UV-326, UV-327, UV-329
150	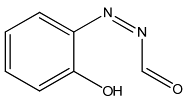<br>Found in: UV-P	163	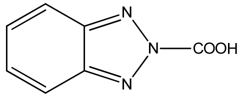<br>Found in: UV-328	182	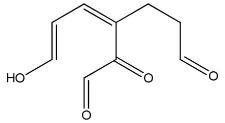 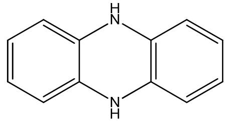 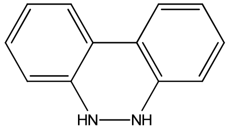<br>Found in: UV-P
151	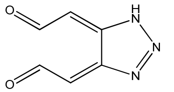 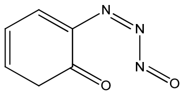<br>Found in: UV-P, UV-326	167	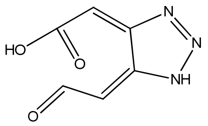 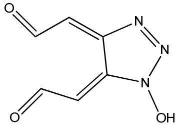<br>Found in: UV-P, UV-326, UV-328	191	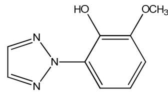<br>Found in: UV-P, UV-328

Table S11 continued

196	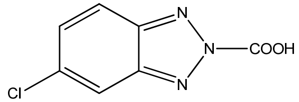 Found in: UV-326, UV-327	275	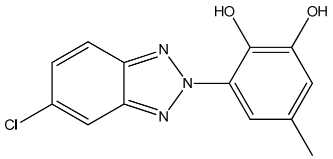 Found in: UV-326	301	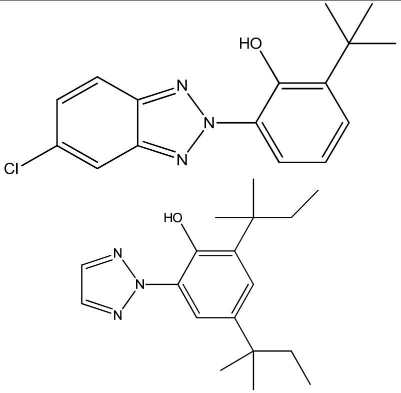 Found in: UV-326, UV-328
231	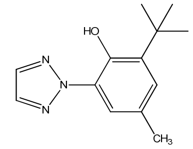 Found in: UV-326, UV-327	283	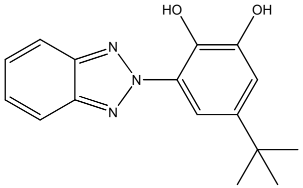 Found in: UV-327	317	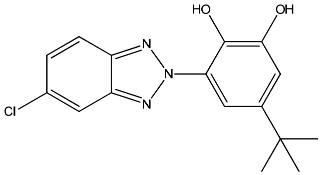 Found in: UV-326, UV-327
241	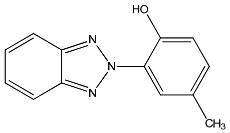 Found in: UV-P	297	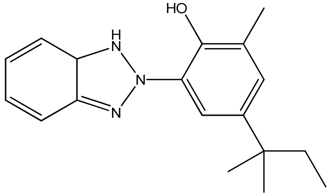 Found in: UV-328	347	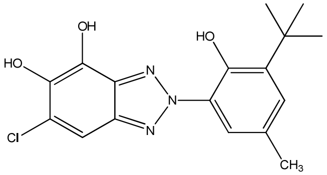 Found in: UV-326, UV-327
273	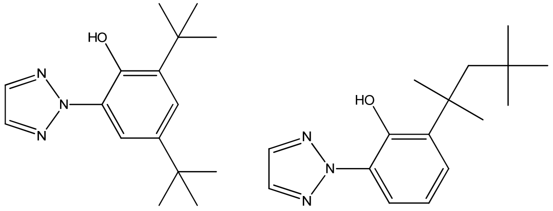 Found in: UV-327, UV-329				

References

1. Luukkonen, T. New Adsorption and Oxidation-Based Approaches for Water and Wastewater Treatment. Studies Regarding Organic Peracids, Boiler-Water Treatment, and Geopolymers, University of Oulu Graduate School; University of Oulu, Faculty of Science, 2016.
